# Occupation and SARS-CoV-2 infection risk among workers during the first pandemic wave in Germany: potential for bias

**DOI:** 10.5271/sjweh.4052

**Published:** 2022-10-01

**Authors:** Martie van Tongeren, Sarah Rhodes, Neil Pearce

**Affiliations:** 1 School of Health Science, University of Manchester, Manchester, UK; 2 London School of Hygiene and Tropical Medicine, London, UK

We read with great interest the paper by Reuter et al (1) on the differences in risk of SARS-CoV2 infection by occupation during the first pandemic wave in Germany. Occupation has been linked with differential risks of infection (2, 3) as well as severe disease and death (4, 5). Hence, this is a potentially very important paper, advancing the evidence in relation to occupational risk factors for infection.

This study makes use of an existing cohort (the German National Cohort – NAKO), with data from over 100 000 workers who were employed or self-employed and completed a COVID-19 questionnaire. SARS-CoV2 infection was assessed through a self-reported positive PCR test carried out in a doctor’s practice, test centre or in a hospital. The main analyses used a Poisson regression model to obtain incidence rates of infection by occupation, both crude and analyses adjusted for potential confounding factors (sociodemographic and employment related factors) were carried out.

Based on the results of the analyses, the authors conclude that (i) there were relatively high infection rates in healthcare and personal services but also in business management and business services, (ii) there were relatively low infection rates in manufacturing and production related occupations, and (iii) there was an inverse social gradient between occupational position and risk of infection, with higher risk in occupations with advanced tertiary degrees/managers.

Like other studies, these analyses found relatively high infection rates in essential occupations. However, important differences with other studies included the inverse social gradient and the relatively high infection rates in occupations with management responsibility and requiring higher degrees. The authors postulated a possible explanation for this finding, stating that managers in Germany may be at higher risk due to recreational ski trips.

Although this may well be a partial explanation, we argue that there is a more likely explanation for the high rates in higher educated people and those working in the healthcare sector. These groups are more likely to have been tested, particularly during the early stage of the pandemic, compared to other occupations such as those working in manufacturing and production-related occupations. This could be due to differential access to testing due to employer requirements or financial restraints (especially at times when tests were not free for all in Germany 1) or different motivations for testing (due to lack of sick pay or self-employment). The authors estimate the infection rates using these positive tests as the numerator and the total cohort population (many of whom have never been tested) as the denominator. Therefore, if there is a differential likelihood of testing between different occupations, this would lead to bias in the results.

It is relatively simple to address this problem by using a test-negative design (6, 7), which is a type of case–control approach where those with a positive test are compared to those who have tested negative (ie, excluding those who have never been tested). This has been widely used as the gold standard method for studying vaccine effectiveness (8) and is increasingly being used to study risk factors for COVID-19 infection.

We would encourage the authors to carry out such analyses and present the results in their response to this letter.

If, as we expect, the high relative risks in those with higher education and/or managers are reduced in these analyses, this would strongly indicate that the reported findings are primarily due to selection bias.
